# Therapeutic management of botulism in dairy cattle

**DOI:** 10.14202/vetworld.2015.1305-1309

**Published:** 2015-11-21

**Authors:** S. Jegaveera Pandian, M. Subramanian, G. Vijayakumar, G. A. Balasubramaniam, K. Sukumar

**Affiliations:** 1 Division of Livestock and Fisheries Management, ICAR Research Complex for Eastern Region, Patna, Bihar, India; 2 Department of Veterinary Clinical Medicine, Veterinary College and Research Institute, Namakkal- 637 002, Tamil Nadu, India; 3 Department of Veterinary Pathology, Veterinary College and Research Institute, Namakkal- 637 002, Tamil Nadu, India; 4 Department of Veterinary Public Health and Epidemiology, Veterinary College and Research Institute, Tirunelveli- 627 001, Tamil Nadu, India

**Keywords:** botulism, cattle, neurotoxin, paralysis, therapy

## Abstract

**Aim:**

To report the successful recovery of few dairy cattle from botulism in response to a modified therapeutic strategy.

**Materials and Methods:**

Seventy four naturally-occurring clinical cases of bovine botulism encountered during the period of 2012-2014 which were confirmed by mouse lethality test became material for this study. Affected animals were made into three groups based on the treatment modifications made during the course of study.

**Results and Discussion:**

With the modified therapeutic regimen, 17 animals recovered after 7-10 days of treatment. Clinical recovery took 2-30 days. Animals which were not given intravenous fluid and calcium recovered uneventfully. Cattle which were already treated with intravenous fluids, calcium borogluconate, and antibiotics did not recover. They were either died or slaughtered for salvage.

**Conclusion:**

In cattle with botulism, administration of Vitamin AD_3_E and activated charcoal aid the clinical recovery. Besides, strictly avoiding anti-clostridial antibiotics, fluid therapy, and calcium therapy may facilitate the clinical recovery. Upon fluid administration, the pulmonary congestion existed in the ailing cattle might have worsened the anoxia. Administration of antibiotics like penicillin, aminoglycosides, and tetracyclines further worsen the neuronal paralysis by increasing the availability of botulinum neurotoxin. Cattle in early botulism have fair chances of recovery with the modified therapy.

## Introduction

Botulism is an exotoxin-induced flaccid paralysis affecting animals and human. Cattle are no exception for this peripheral neuronal paralysis. Being a bio-warfare agent and the most potent toxin known to date, botulinum neurotoxin (BoNT) plays havoc in living beings. The causative agent, *Clostridium botulinum* is a ubiquitous soil-borne pathogen, prefers to grow well in decaying organic matter [1]. There were few sporadic and unconfirmed reports of bovine botulism from India also [2,3]. Infrequent isolations of organisms are reported from many regions. From aquatic environments, Types C and D have been isolated and reported [4,5]. In the recent past, sporadic incidences and cluster outbreaks of poultry litter associated bovine botulism have been reported from different parts of the world [6,7].

The botulism usually ends in fatality as the neuronal paralysis cannot be reversed by available therapeutic options. Conventionally, administration of antitoxin was suggested as the first line of management. Human botulism cases have been treated successfully with antitoxin, mechanical ventilation, and other symptomatic therapeutic measures [8]. But, the availability of antitoxin in developing countries is still a distant dream. However, antitoxin therapy would be successful if it is initiated before the toxin reaches motor-end plate [9].

Many researchers attempted to save cattle from botulism through symptomatic management. Rare reports of clinical recovery are also available in literature. In this study, 74 clinical cases of bovine botulism were encountered and treated during the period of March 2012-February 2014. Among them, 17 animals recovered clinically following a modified therapeutic strategy. The details of therapy and sequel are elaborated and discussed herewith.

## Materials and Methods

### Ethical approval

With due approval from Institute Animal Ethics Committee, KMCH College of Pharmacy, Coimbatore, Tamil Nadu, the mouse lethality test was conducted.

### Sample collections

Seventy four dairy cattle reported/referred to Teaching Veterinary Clinical Complex, Veterinary College and Research Institute, Namakkal with clinical signs of botulism were included for this study. All these animals were owned by different farmers and reared at different locations. Animals were at different stages of the production cycle and maintained under semi-intensive system. After a detailed clinical examination, clinical materials including blood, feces, and rumen fluid were collected and routine hematological and serum biochemical parameters were estimated [10,11]. Rumen fluid, fecal samples, and environmental samples (poultry droppings, swabs collected from carcasses, and soil swabs) were subjected for bacteriological culture and mouse lethality test.

### Mouse lethality test

Swiss albino mice of either sex weighing 20-30 g were inoculated with serum, inoculums prepared from dung and rumen liquor samples of the affected animals. Serum and culture supernatants were injected intra-peritoneally as such. But, the dung and rumen fluid samples were prepared as inoculums by the following manner. 1 ml of cold (4°C) gelatin diluent (0.2% gelatin, 0.4% Na_2_PO_4_; pH 6.4) was added to each gram of dung/rumen liquor. It was mixed well to obtain a uniform suspension. The obtained suspension was held at 4°C for 30 min. Clarification of supernatant was done in a refrigerated centrifuge at 12,000 rpm for 20 min. Trypsin solution (0.25 ml of 1:250 solution) was added to each milliliter of clarified supernatant. Inoculation involves single intraperitoneal injection of the suspected material (0.5 ml) after proper preparation as inoculums. Inoculated mouse was observed at 1,2, 4, 8, 12, 18, and 24 h interval on the1^st^day and thereafter daily for 4 days (96 h) for the development of ruffling of fur, labored abdominal breathing, weakness of limbs, and total paralysis. The death of inoculated mice after exhibiting signs like ruffling of fur, abdominal breathing, “wasp-waist” appearance, and total motor paralysis confirmed the presence of BoNT [12,13] in the suspected materials. The results of mouse lethality test is given in Table-1.

**Table-1:** Results of mouse lethality test in cattle with botulism.

Samples description (in duplicate)	Number of samples inoculated	Absolute number of samples	Positive (%)	Negative (%)
Control I-healthy cattle rumen liquor with gelatin diluent	1	2	-	100
Control II - healthy cattle dung sample only	1	2	-	100
Control III- gelatin diluent	1	2	-	100
Control IV- healthy cattle serum	1	2	-	100
Rumen fluid - affected cattle	12	24	66.66	33.33
Dung sample - affected cattle	12	24	83.33	16.66
Serum - recumbent cattle	12	24	8.33	91.66
Total	40	80		

### Treatments

Treatment for the Group I (n=34) was not instituted by the authors as those animals were already treated by the practicing veterinarians with a variety of drugs and regimen and referred to the authors. On reception of the case, they were treated with intravenous isotonic fluids and activated charcoal at 1 g/kg BW P.O. The adopted treatment strategy for Group II (n=13) was the administration of intravenous isotonic saline as per the degree of dehydration and activated charcoal and B-complex vitamin injections [14]. The treatment for the Group III (n=27) was modified based on the response to therapy and necropsy findings of Groups I and II. The treatment regimen for Group III was as follows. Activated charcoal (at 1 g/kg body weight PO) for 2 consecutive days; Vitamin AD_3_E (commercial product composition in each ml: Vitamin A: 2.5 lakhs IU; Vitamin D_3_: 25,000 IU; Vitamins E: 100 mg; biotin-15mcg) at 10 ml IM for a period of 4-5 days. Antibiotics and intravenous fluids were strictly avoided. Along with them, trace mineral bolus at 1 PO s.i.d (composition: copper 250 mg; zinc 500 mg; selenium 3 mg; cobalt 60 mg; iodine 50 mg; manganese 600 mg, Vitamin A 8000 I.U; and Vitamin E 500 I.U) and probiotic bolus containing *Saccharomyces cerviciae* at 1 b.i.d P.O were given for a period of 7-10 days. Besides nursing, the response to treatment and clinical recovery characteristics were recorded for each group.

## Results

Anamnesis of Group I (n=34) animals revealed that they were treated with intravenous solutions containing calcium borogluconate and magnesium, antibiotics (gentamicin, streptomycin-penicillin combination, and enrofloxacin), intravenous fluids (isotonic saline, dextrose [5%], dextrose [10%], and Ringer’s lactate), and meloxicam. The entire group I animals were treated for a period of 2-3 days by practitioners.

The posture of the animals before initiation of treatment is given in Table-2. Clinical signs manifested were abdominal breathing, scanty unformed dung, tripping gait, rumen atony, sternal recumbency progressing to lateral recumbency in a span of 4-12 h, frequent pedaling of limbs, and reduced retractile strength of tongue and salivation. The rectal temperature, heart rate, and other vital parameters were within the physiological range although there were insignificant changes associated with the stage of disease. Hematology and serum biochemical analysis revealed erythrocytosis, increased hematocrit, leukocytosis, mild neutrophilia, lymphocytosis, and monocytosis but inconsistently in each animal. Hemoglobin concentration, mean corpuscular volume, mean corpuscular hemoglobin (MCH), and MCH concentration were unremarkable. A reduction in the blood pH was observed. Elevated serum urea nitrogen and creatinine were observed in ailing animals. Except hypokalemia, all other biochemical parameters were within the physiological range. The results of mouse lethality test are given in Table-1.

**Table-2:** The posture of clinical cases of bovine botulism at the time of presentation.

Groups	Standing	Sternal recumbency+ alert mentation	Sternal recumbency+ depressed mentation	Lateral recumbency
I (n=34)	2	27	1	4
II (n=13)	0	13	0	0
III (n=27)	11	12	4	0

**Figure-1:**
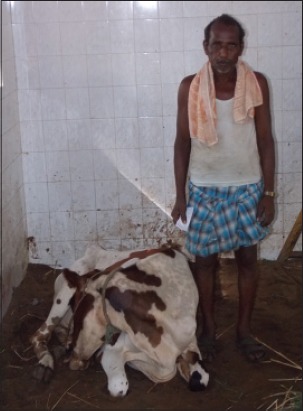
Sternal recumbency in a cow with botulism before treatment; posture resembled second-stage hypocalcemia.

**Figure-2:**
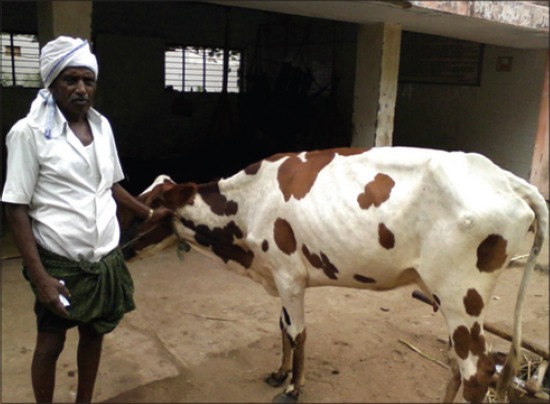
Cow recovered after 13 days of treatment and nursing.

None of the animals in Group I and II recovered. Moribund animals were either salvaged for slaughter or died after a period of 2-10 days. Among the Group III animals, 17 animals recovered (Figures-1 and 2). Recovery from botulism was characterized by the quality of dung returning to normalcy with colon marks, resumption of voluntary feed intake, reduction in hind limb tripping, restored rumen motility, and rumination. Duration for recovery and the course of treatment ranged from 2 to 30 days and 7 to 10 days, respectively.

## Discussion

The BoNT remains to be the most potent neurotoxin until date for all living beings. The degree of susceptibility of various species is reviewed at length by Num and Useh [15]. Anamnesis of previous treatment given to the recumbent animals revealed that 34 (45.95%) cattle were treated with intravenous calcium borogluconate with magnesium solutions, antibiotics, fluids, and anti-inflammatory drugs. Many practitioners opted for intravenous calcium as botulism mimicked hypocalcemia and other metabolic downers which were enlisted as differential diagnoses for botulism [16]. But, intravenous calcium therapy in botulism was of no use as absorption and dissemination of toxin is favored by calcium [17]. Clinical signs were manifestations of peripheral neuronal flaccid paralysis caused by BoNT. But, laboratory findings were neither pathognomonic nor assuming any diagnostic significance in this study. Less significant hematological changes were reported by many authors [6,14,16].

In this study, cattle which had already been treated intravenously with calcium and magnesium containing solutions did not recover. Death or salvage for slaughter was the sequel after calcium borogluconate administration in cattle suspected for botulism [18]. Some authors have reported recovery of cows from botulism after 1 week [19,20]. In this study, 17 animals recovered over a period of 2-30 days. In Holstein cows, as stated by Martin, the average duration of illness was 4 days (3-14 days). Besides, clinical recovery was noticed after 7 days and weakness persisted up to 1 month [16].

**Figure-3:**
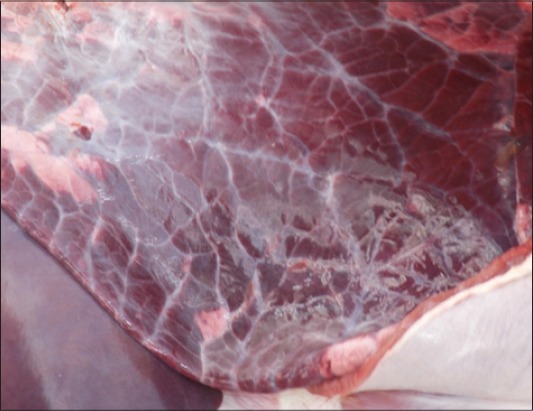
Severe pulmonary congestion; liver is placed distally for comparison.

Many researchers attempted symptomatic management of cattle with botulism [21]. Jean *et al*. used injectable Vitamin E and selenium and isotonic fluid therapy without much success [17]. In this study, Group III cattle with botulism were treated with activated charcoal and Vitamin AD_3_E injections (10 ml IM) daily. No antibiotic was used in Group III as antibiotics like aminoglycosides, tetracyclines, and procaine penicillin tend to worsen the flaccid paralysis caused by BoNT [14]. As a palliative measure, oral administration of activated charcoal, sodium sulfate, and subcutaneous administration of neostigmine were the treatment adoptedby Senturk and Cihan [22]. Early cases in standing posture with restlessness and sternal recumbency with alert mentation were treated successfully. One pregnant cow recovered after 30 days and calved successfully. Kummel *et al*. (2012) reported maintenance of pregnancy and recovery from botulism in a cow [20]. Despite contradicting the recommendations of intravenous fluid administration by earlier researchers, symptomatic management adopted in this study aided the recovery from botulism [14]. This contradiction could be either due to the previous treatment with antibiotics or compromised pulmonary ventilation caused by respiratory paralysis. This observation was very well supported by the presence of severe pulmonary congestion in necropsies (Figure-3). Nevertheless, as reported by many authors,use of antibiotics like penicillins and aminoglycosides further deteriorated botulism and administration of intravenous calcium did not favor recovery in botulism as evidenced by the response in Group I animals [14].

## Conclusions

Fatal botulism in cattle could be managed if treated at the early stage. Early signs like tripping gait, passing unformed dung, abdominal breathing, and recumbency should bring botulism in differential diagnoses. Cattle which assumed lateral recumbency were found to be unsuitable for therapeutic management. Although successful, the modified therapy cannot be claimed superior to antitoxin administration as the latter was not attempted in this study. It is concluded that symptomatic management with activated charcoal, Vitamin AD_3_E, microminerals, and probiotics supplementation bring out good clinical recovery in cattle with botulism. Based on the response to therapy, it can be inferred that intravenous fluid therapy did not aid the clinical recovery in these animals as the pre-existing respiratory paralysis and consequent pulmonary congestion aggravated the hypoxia. It is again proved that use of anti-clostridial antibiotics will further reduce the chance of recovery from botulism.

## Authors’ Contributions

SJP: Experimental design, execution, manuscript preparation and correspondence; MS and GV: Experimental design and guidance; GAB: Contributed in clinical-pathology part; KS: Contributed in microbiological and toxicological part of the study. All authors read and approved the final manuscript.
